# Remotely Supervised Home-Based tDCS in Treatment-Resistant Depression: Feasibility, Effectiveness and EEG Biomarkers of Response

**DOI:** 10.21203/rs.3.rs-8709698/v1

**Published:** 2026-01-29

**Authors:** Rubén Romero-Marín, Sergi López-Rodríguez, Sara Lakis-Granell, Edgar Buloz-Osorio, María Cabello-Toscano, Mikel Urretavizcaya-Sarachaga, Maria del Pino Alonso-Ortega, Mohit Chopra, Javier Solana-Sánchez, Joan Camprodon, Álvaro Pascual-Leone, David Bartrés-Faz, Davide Cappon, Gabriele Cattaneo

**Affiliations:** Institut Guttmann Institut Universitari de Neurorehabilitació adscrit a la UAB, Badalona, Spain; Institut Guttmann Institut Universitari de Neurorehabilitació adscrit a la UAB, Badalona, Spain; Bellvitge University Hospital-Institute of Neurosciences of the University of Barcelona UB neuro-Bellvitge Biomedical Research Institute IDIBELL, Barcelona, Spain; Institut Guttmann Institut Universitari de Neurorehabilitació adscrit a la UAB, Badalona, Spain; Institut d’Investigacions Biomèdiques August Pi i Sunyer (IDIBAPS), Barcelona, Spain; Bellvitge University Hospital-Institute of Neurosciences of the University of Barcelona UB neuro-Bellvitge Biomedical Research Institute IDIBELL, Barcelona, Spain; Bellvitge University Hospital-Institute of Neurosciences of the University of Barcelona UB neuro-Bellvitge Biomedical Research Institute IDIBELL, Barcelona, Spain; Hinda and Arthur Marcus Institute for Aging Research and Center for Memory Health, Hebrew SeniorLife, Boston, MA, United States; Institut Guttmann Institut Universitari de Neurorehabilitació adscrit a la UAB, Badalona, Spain; Massachusetts General Hospital-Harvard Medical School, Boston, MA, United States; Department of Neurology, Harvard Medical School, Boston, MA, United States; Institut d’Investigacions Biomèdiques August Pi i Sunyer (IDIBAPS), Barcelona, Spain; Department of Neurology, Harvard Medical School, Boston, MA, United States; Institut Guttmann Institut Universitari de Neurorehabilitació adscrit a la UAB, Badalona, Spain

**Keywords:** transcranial direct current stimulation, tDCS, HB-tDCS, treatment-resistant depression, TRD, major depressive disorder, MDD

## Abstract

**Background:**

Remotely supervised home-based transcranial direct current stimulation (HB-tDCS) may expand access to neuromodulation for treatment-resistant depression (TRD), but real-world evidence on candidate biomarkers remains limited.

**Methods:**

In this naturalistic pre–post study, 40 adults with major depressive disorder and inadequate response to ≥ 2 treatments were enrolled. After MRI/clinical screening, 2 were excluded for potential stimulation contraindications and 5 did not start or discontinued within the first sessions for personal reasons unrelated to stimulation. 33 participants completed a 6-week semi-supervised HB-tDCS protocol (42 sessions, 30 min, 2 mA; anode F3/cathode F4). Baseline and post-treatment assessments included clinician-rated and self-rated depression (MADRS, QIDS-SR16, BDI-II), global cognition (MoCA), quality of life (Q-LES-Q-SF), and neurophysiological markers (resting-state EEG and TMS-EEG). Feasibility, technical incidents, and adverse effects were recorded after each session.

**Results:**

Completers delivered 1,219/1,386 scheduled sessions (88%). Depressive symptoms decreased from baseline to post-treatment (MADRS − 24%, QIDS-SR16 − 15%, BDI-II − 12%; all p≤.002). Sixteen of 33 (48.5%) achieved ≥ 25% MADRS reduction (partial response), including 5 (15.2%) with ≥ 50% reduction. MoCA improved modestly, whereas Q-LES-Q-SF did not change significantly. Adverse effects were mostly mild/moderate (e.g., tingling, headache, scalp dryness), and no stimulation-related serious adverse events occurred. Exploratory analyses suggested higher baseline left-frontal alpha (F3) relative power and TMS-EEG evoked potentials in the left DLPFC (P30, P180) differentiated responders from non-responders.

**Conclusions:**

An intensive, semi-supervised HB-tDCS program was feasible and well tolerated in TRD and was associated with clinically meaningful symptom reductions. Candidate EEG/TMS-EEG markers warrant replication in controlled trials.

## 1 Introduction

Major depressive disorder (MDD) is a highly prevalent and disabling condition, affecting an estimated 332 million people worldwide and ranking among the leading causes of years lived with disability (Institute of Health Metrics and Evaluation, 2021; World Health Organization, 2025). Despite the availability of multiple pharmacological and psychotherapeutic options, 20–40% of patients fail to achieve satisfactory improvement and develop treatment-resistant depression, typically defined by non-response to at least two adequate therapeutic trials (Fava et al., 2007; Nemeroff, 2007; Rush et al., 2006). This group carries a disproportionate burden of functional impairment, healthcare use, and suicide risk, underscoring the need for alternative, mechanism-informed interventions.

Contemporary models of MDD emphasize dysfunction in large-scale brain networks rather than isolated regions. Converging structural and functional imaging studies describe hypoactivation and hypoconnectivity of the left dorsolateral prefrontal cortex (L-DLPFC), alongside relative hyperactivity of right prefrontal and limbic structures, particularly the subgenual cingulate cortex (SGC) (Fox et al., 2012; Grimm et al., 2008; Koenigs and Grafman, 2009). Positron emission tomography studies consistently report SGC hyperactivity that is anticorrelated with DLPFC activity, suggesting impaired top-down regulation of negative affect and stress responses (Hadas et al., 2019; Luscher et al., 2011; Stolz et al., 2023). These findings support the view of depression as a disorder of network-level imbalance, consistent with emerging circuit-based taxonomies of mood and anxiety disorders (Lynch et al., 2024; Palacek et al., 2025; Williams, 2017, 2016).

Non-invasive brain stimulation (NIBS) techniques, such as repetitive transcranial magnetic stimulation (rTMS) and transcranial electrical stimulation (tES), offer a way to modulate these circuits causally. Stimulation of the DLPFC can influence distributed networks that include the SGC and other limbic hubs, leading to clinically meaningful antidepressant effects even in treatment-resistant cases (Downar et al., 2024; Schlaepfer et al., 2010; Valero-Cabré et al., 2008). rTMS targeting the L-DLPFC is now an established treatment for medication-resistant depression, with response rates of approximately 60% and remission rates of around 30% in large trials and guideline-based practice (Blumberger et al., 2018; George, 2010; O’Reardon et al., 2007; Pascual-Leone et al., 1996; Perera et al., 2016). Transcranial direct current stimulation (tDCS), which delivers weak direct currents through scalp electrodes, has also demonstrated moderate antidepressant efficacy, particularly when combined with pharmacotherapy or psychotherapy, typically using anodal stimulation over the L-DLPFC (F3) and a contralateral or supraorbital cathode (Nikolin et al., 2023; Razza et al., 2020; Ren et al., 2025).

However, conventional NIBS delivery is constrained by the need for frequent, clinic-based sessions over several weeks. For many patients with treatment-resistant depression (TRD), repeated travel to specialized centers is logistically difficult and costly, limiting access, adherence, and scalability. Home-based tDCS (HB-tDCS) has emerged as a promising alternative: by enabling patients to self-administer stimulation with remotely supervised devices, HB-tDCS can reduce treatment burden while maintaining safety and procedural integrity (Cappon et al., 2022; Palm et al., 2018). Recent feasibility and randomized trials in MDD suggest that remotely supervised HB-tDCS is acceptable, safe and capable of producing clinically relevant symptom improvements (Alonzo et al., 2019; Borrione et al., 2020, 2024; Castelo-Branco and Fregni, 2020; Romero-Marín et al., 2026a; Woodham et al., 2024).

Moreover, recent pivotal randomized, sham-controlled trials have provided regulatory-level evidence supporting HB-tDCS for MDD, leading to FDA approval of the Flow tDCS system for home treatment of moderate-to-severe MDD and the Neurolief system for adjunctive at-home neuromodulation in TRD, based on clinically meaningful symptom improvements demonstrated in these trials (Borrione et al., 2024; Carpenter et al., 2025).

Despite growing evidence and recent regulatory approvals, important challenges remain for the implementation and optimization of home-based neuromodulation in TRD. In particular, it is still unclear whether scalable neurophysiological measures can reliably differentiate responders from non-responders and support treatment monitoring in routine-care settings. In parallel, there is no consensus on key protocol parameters—including optimal dosing (number and spacing of sessions), stimulation targets/montages, and patient characteristics that may predict benefit. Addressing these gaps is essential to inform implementation pathways and to move beyond one-size-fits-all protocols toward data-driven stratification and personalization.

In parallel, there is growing recognition that depression is a heterogeneous syndrome comprising partially dissociable symptom dimensions (e.g., dysphoric vs. anxiosomatic features) that map onto distinct brain circuits. Network-based targeting work has shown that stimulation sites functionally anticorrelated with the SGC (“anti-subgenual” targets) preferentially modulate dysphoric symptoms, whereas stimulation of more dorsal L-DLPFC and dorsomedial prefrontal regions preferentially impacts anxious and somatic symptoms (Fox et al., 2012; Siddiqi et al., 2020; Siddiqi and Fox, 2024; Williams, 2017, 2016). This circuit-level perspective has accelerated interest in neurophysiological biomarkers that could predict or explain treatment response. This issue is particularly salient in light of the recent FDA approvals of home-based neuromodulation devices such as Flow and Neurolief, which implement fixed stimulation protocols without individualization of targets or parameters. While it remains unclear whether personalization meaningfully improves clinical outcomes, adequately powered datasets linking symptom dimensions, circuit-level engagement, and treatment response are required to empirically address this question.

Resting-state EEG and TMS-EEG are particularly attractive in this context. Resting EEG measures such as frontal alpha power and asymmetry have long been explored as correlates of affective style and depression, and recent systematic reviews highlight their potential as predictors of treatment response to NIBS (Roelofs et al., 2021; Romero-Marín et al., 2026b; Stolz et al., 2023; Widge et al., 2019). TMS-EEG, which combines focal magnetic pulses with concurrent EEG recording, provides a direct index of cortical excitability and inhibition, as well as network-level propagation, through TMS-evoked potentials (TEPs) and global mean field measures. Specific TEP components (e.g., P30, N45, N100, P180) have been proposed as markers of excitatory–inhibitory balance and plasticity (Premoli et al., 2014; Strafella et al., 2022), and emerging data suggest that baseline TEP profiles and their modulation over treatment may predict outcomes in rTMS and theta-burst protocols for TRD (Eshel et al., 2020; Hadas et al., 2019; Strafella et al., 2023; Voineskos et al., 2021).

Yet, few studies have integrated intensive HB-tDCS with EEG in a naturalistic TRD sample. Key open questions include: (i) whether a semi-supervised, daily HB-tDCS regimen is feasible and safe in patients with long-standing, treatment-resistant depression; (ii) what magnitude of clinical benefit can be expected under real-world conditions; and (iii) whether prefrontal EEG and TMS-EEG marker, particularly resting alpha power and DLPFC TEPs at dysphoric and anxiosomatic targets, can differentiate responders from non-responders or track clinical change (Cappon et al., 2022; Palm et al., 2018; Razza et al., 2020; Strafella et al., 2022; Widge et al., 2019). Given the limited focality of home-based sponge tDCS, we treat the dysphoric and anxiosomatic DLPFC sites as TMS-EEG probe locations rather than distinct stimulation targets. Addressing these questions would help clarify the clinical utility and mechanistic underpinnings of HB-tDCS and inform future precision neuromodulation strategies.

We hypothesized that (i) the HB-tDCS protocol would prove feasible and well tolerated; (ii) patients would show clinically meaningful reductions in dysphoric and anxiosomatic depressive symptoms over 6 weeks; and (iii) baseline and/or treatment-related changes in prefrontal EEG and TEP measures would differentiate clinical responders from non-responders, providing candidate biomarkers for future personalized HB-tDCS interventions.

## 2 Methods

In this study, we conducted a semi-supervised HB-tDCS program in adults with treatment-resistant MDD (TRD). Participants underwent comprehensive clinical, cognitive, and neurophysiological assessments at baseline and post-treatment, including clinician- and self-rated depression, global cognition, quality of life, resting-state EEG, and TMS-EEG.

### Participants

Patients eligible for this study were adults (18 years or older) diagnosed with Major Depressive Disorder (MDD) according to the DSM-V-TR (American Psychiatric Association, 2022), who had tried at least two previous treatments without achieving the expected outcomes. Additional inclusion criteria required a minimum score of 20 on the Montgomery–Åsberg Depression Rating Scale (MADRS), consistent with inclusion thresholds used in prior home-based tDCS trials (Alonzo et al., 2019), and confirmation by their primary psychiatrist that they were stable enough to participate safely. All participants were screened to ensure they had no contraindications to tDCS, MRI, or TMS procedures (such as cranial metal implants, pacemakers, or infusion pumps) (Rossi et al., 2021) and did not present other psychiatric diagnoses, neurological conditions, substance abuse, or pregnancy. Patients taking antidepressant medication were permitted provided their dosage had been stable for at least one month before entering the trial. All eligibility criteria were reviewed and confirmed by the study psychiatrist. Prior to participation, all eligible individuals provided explicit written informed consent after receiving detailed information about the study procedures and possible risks. The protocol was approved by the Ethics and Clinical Research Committee of the Catalan Hospitals Union (Comitè d’Ètica d’Investigació amb medicaments (CEIm) de la Fundació Unió, CEI 22/105), and registered as a clinical trial at clinicaltrials.gov (NCT05930509).

Finally, a total of 40 participants diagnosed with TRD were recruited through referrals from the outpatient depression clinic of the Bellvitge University Hospital and the Guttmann Institute (see Table 1).

**Figure 1:**
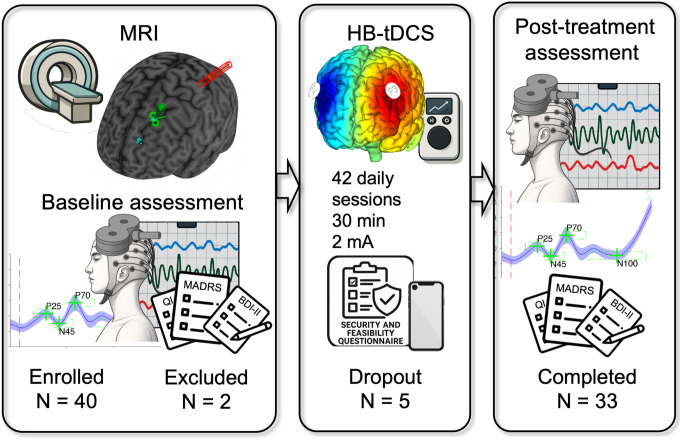
Study design. Overview of the study workflow including MRI screening and neuronavigation setup, baseline clinical and neurophysiological assessments, a 6-week semi-supervised home-based tDCS intervention, and post-treatment clinical and neurophysiological reassessment.

First, participants attended an initial interview to assess eligibility criteria and to receive detailed information about the study procedures. Those who met the inclusion criteria and agreed to participate provided written informed consent. During this first visit, we also scheduled an MRI scan. The MRI served two purposes: to ensure participants did not have any conditions incompatible with tDCS and TMS stimulation, and to acquire brain images needed for configuring the neuronavigation system. At the subsequent visit, we conducted the baseline assessment, which included cognitive and behavioral evaluations of depressive symptoms, resting-state EEG, and TMS-EEG recordings. Finally, participants and their companions received training on how to use the tDCS device. They completed a supervised practice session, after which they were provided with the device to continue the treatment at home (similar to Cappon et al., 2023; see Fig. 1).

### Semi-supervised Home-Based tDCS protocol

The intervention consisted of 42 consecutive daily sessions (7/7 days) over 6 weeks, each 30 minutes at 2 mA, with no taper phase using a tDCS device (Sooma tDCS^™^ device, Oy, Helsinki, Finland). This daily regimen follows recent intensive schedules reported in the literature, for example, 4-week daily dosing (Ruffini et al., 2024), while extending to 6 weeks to maximize cumulative exposure.

#### Stimulation targets and montage

The anode was positioned at F3 (left DLPFC) and the cathode at F4 (right DLPFC) according to the international 10–20 EEG system. Electrode placement was standardized using cap landmarks and patient-specific photos taken at training to ensure reproducibility across sessions. Circular electrodes with 6-cm-diameter saline-soaked sponge pads were used.

#### Set-up, training, and semi-supervision

Participants received structured training (patient and companion) on device use, skin preparation, electrode positioning, and safety checks (Cappon et al., 2023). Sessions were self-administered at home, independently or with companion assistance. After each session, participants completed an online survey documenting feasibility (completion, interruptions/restarts, support needed) and adverse events. The research team asynchronously reviewed the uploaded session logs and survey data the following day; if issues were detected (e.g., repeated high-impedance restarts, AE flags), the participant was contacted by phone to troubleshoot. Additionally, weekly telephone calls were scheduled to review progress, capture AEs not reported in surveys, and address practical difficulties (e.g., fit/impedance). Device impedance checks were mandatory before current ramp-up; sessions were restarted automatically when out-of-range impedance was detected. After completing the 6-week course, participants returned for post-treatment assessments identical to baseline.

### Depression symptoms and cognitive assessment

Patients underwent a comprehensive psychiatric evaluation including the Montgomery–Åsberg Depression Rating Scale (MADRS), the Beck Depression Inventory (BDI-II), and the Quick Inventory of Depressive Symptomatology (QIDS-SR16). Cognitive performance was assessed using the Montreal Cognitive Assessment (MoCA). All evaluations were administered at baseline and post-treatment to quantify change over the 6-week intervention period.

To characterize clinical improvement, percentage change from baseline was calculated for each scale. Participants showing a ≥ 25% reduction in depressive-symptom scores were classified as partial responders, those with a ≥ 50% reduction as responders, and those with < 25% change as non-responders.

### Resting state EEG and TMS-EEG

Patients underwent a neurophysiological assessment at baseline and post-treatment, which included resting-state EEG recordings with eyes open and eyes closed, each lasting five minutes. To minimize auditory interference, patients wore earplugs during EEG acquisition. EEG data were collected using a 64-channel EasyCap system from BrainVision. For the TMS-EEG protocol, we employed a MagPro stimulator (MagVenture Inc.) with neuronavigation performed using Polaris Viewer, guided by each patient’s 3D T1-weighted MRI to compute target coordinates. Earplugs and additional white noise were used to mask external sounds during TMS-EEG. Stimulation targets were selected based on previous studies (Siddiqi et al., 2020) to assess neurophysiological markers of depressive symptoms, with the dysphoric target located near the “anti-subgenual” region at MNI coordinates [−32, 44, 34], and the anxiosomatic target positioned close to the conventional 5-cm site used in early TMS trials for depression at MNI coordinates [−37, 22, 54]. All participants received stimulation at both targets (within the same assessment session), with 120 single pulses delivered per target using a figure-eight MCF-B65 coil; inter-stimulus intervals were randomly jittered between 3–5 s. The resting motor threshold (RMT) was determined over the left motor cortex prior to baseline TMS-EEG measurements, defined as the minimum stimulus intensity required to elicit motor evoked potentials greater than 50 μV in at least 5 out of 10 trials (Rossini et al., 1994).

To preprocess the EEG data, we used MATLAB (MathWorks Inc.) and the EEGLAB toolbox with the ICLabel plugin. Continuous resting-state EEG recordings were imported from BrainVision files and electrode locations were assigned using the BESA 10–5 template. Signals were down-sampled to 250 Hz and high-pass filtered at 1 Hz to stabilize the baseline and remove slow drifts. Line noise at 50 Hz was removed using the CleanLine plugin (2 Hz bandwidth, 4-s windows, 1-s step, p = 0.01). Noisy or unstable channels were identified by combining Artifact Subspace Reconstruction (ASR) with visual inspection and were reconstructed using spherical spline interpolation. Data were then re-referenced to the common average to obtain a topographically neutral reference.

For resting-state analyses, continuous data were segmented into non-overlapping 2-s epochs defined by artificial events spaced every 2 s. Artifactual epochs were rejected using a mixed automatic–manual procedure: probability (3.5 SD at the channel level and 3 SD globally), kurtosis (5 SD per channel and 3 SD globally), and an amplitude criterion of ± 100 μV over the entire epoch, excluding frontopolar channels (e.g., Fp1/Fp2) from the amplitude test to avoid blink-related bias. Labeled epochs were visually inspected and removed when necessary. In eyes-open segments, when specified, we applied independent component analysis (ICA) and classified components with ICLabel, rejecting those corresponding to non-neural artefacts (ocular, muscle, or electrode noise). Cleaned data were then used to compute power spectral density (1–80 Hz) and relative band power (delta, theta, alpha, beta, low and high gamma), as well as frontal alpha asymmetry indices and derived spectral ratios.

TMS-EEG data were processed in MATLAB using EEGLAB, FastICA, and the TMS-EEG Signal Analyzer (TESA). BrainVision files were imported with BESA 10–5 electrode coordinates and segmented into epochs from − 1 to 2 s relative to each TMS pulse, with a long baseline correction applied from − 900 to − 100 ms. Channel inspection and rejection were performed on a copy of the data filtered for visualization (57–63 Hz notch, 1–50 Hz band-pass; 4th-order Butterworth), and marked channels (maximum three per recording) were removed from the unfiltered dataset. The early TMS artefact was removed using temporal suppression (zero-padding) implemented in TESA; for each participant we estimated the minimal latency at which the mean signal amplitude across channels and trials dropped below 150 μV (typically ~ 14 ms), and this latency was visually verified.

Epoch rejection combined automatic statistics and visual review. Automatic labelling used probability (3.5 SD per channel, 3 SD globally), kurtosis (5 per channel, 3 globally), and an amplitude threshold of ± 100 μV, excluding frontopolar electrodes (Fp1, Fpz, Fp2, AF7, AF8). The evaluation window was restricted to − 0.5 to 1 s around the pulse, omitting the 0–50 ms interval to avoid contamination from residual TMS artefact. Only epochs confirmed as contaminated were removed from the unfiltered signal. Artefact separation followed a two-step ICA strategy: a first FastICA decomposition (after PCA reduction to 40 components) was used to identify and suppress early TMS-induced muscle activity; missing samples due to suppression were linearly interpolated, followed by 48–52 Hz notch, 1–100 Hz band-pass filtering, and re-referencing to the average. Data were then re-epoched to − 0.5 to 1 s and a second FastICA (PCA to 38 components) was run. Components were classified as TMS artefact, blinks, horizontal eye movements, muscle, or electrode noise based on temporal and spectral criteria, and those labelled as artefactual were removed. Finally, a 50 Hz low-pass filter (4th-order Butterworth) was applied, and previously removed electrodes were reconstructed by spherical interpolation to restore full topography. From the cleaned signal, TMS-evoked potentials (TEPs) were averaged in the 0–300 ms window separately for each stimulation condition (i.e., pulses delivered to the dysphoric vs the anxiosomatic target), and standard components (P30, N45, P60, N100, P180, etc.) as well as global mean field amplitude (GMFA) were computed, with TEP components quantified at F3 using the TESA toolbox (Rogasch et al., 2017). The GMFA time-series was extracted for each participant and condition and subsequently normalized by dividing it by the mean GMFA value during the baseline period, defined as − 450 to − 50 ms relative to the TMS pulse onset. Peaks in the GMFA time-series were identified automatically using a custom MATLAB script. Peak detection thresholds were defined as the maximum of either (i) the mean baseline GMFA plus two standard deviations or (ii) the maximum GMFA value observed during the baseline period. Only peaks occurring within a predefined post-stimulation time window of interest (15–400 ms after the TMS pulse) were retained for further analyses; peaks outside this interval were excluded. To obtain a global measure of cortical reactivity, the area under the curve (AUC) of the normalized GMFA time-series was calculated over the 15–400 ms post-stimulation interval using trapezoidal numerical integration. The GMFA-AUC was selected as an index of overall cortical excitability, consistent with previous TMS-EEG literature (Strafella et al., 2022).

## 3 Results

### Adherence, safety and feasibility

2 out of the 40 participants recruited were excluded after first visit and MRI for potential contraindication for stimulation, including the presence of intracranial metallic clips in one case and relevant medical conditions in the other. Other 5 participants did not initiate or discontinued the intervention after few days/sessions for personal reasons not related to the stimulation.

Feasibility and adherence were evaluated in the 33 patients who completed the 6-week HB-tDCS protocol. For these participants, 42 daily sessions were scheduled (total 1,386 sessions). Of these, 1,219 sessions were successfully completed (87.95%), whereas 167 (12.05%) were not performed, mainly due to personal or time-availability reasons.

All 33 patients (100%) finished the treatment course, and 6 (18.2%) completed the full 42 sessions without missing any session. Two participants skipped sessions because of persistent impedance problems, and 3 additional sessions were delivered (repeated) in two participants to compensate for technical issues.

Technical challenges were frequent but generally minor and manageable at home. Across the 1,386 scheduled sessions, 14 (1.15%) could not be completed due to high impedance, despite repeated attempts. Among completed sessions, 509 (41.76%) were automatically restarted because of impedance warnings before or during ramp-up, and 143 (11.73%) were temporarily interrupted and then resumed without loss of the session. Remote technical support related to impedance was required in only 10 sessions (0.82%), and no session required support because of an adverse event.

At the participant level, 26 of 33 patients (78.8%) reported at least one technical problem and 7 (21.2%) needed remote technical assistance at some point, yet all patients were able to self-administer the treatment throughout the 6 weeks. Importantly, these events were predominantly impedance-related and reflect electrode–skin interface issues (e.g., placement, contact quality, drying/movement) rather than device malfunction.

Regarding overall safety, 18 of the 33 completers (54.55%) reported at least one adverse effect during the intervention, whereas 15 (45.45%) did not report any adverse effect in any session.

Across the 1,219 completed sessions, 350 (28.7%) involved at least one adverse effect, almost all rated as mild or moderate. The most frequently reported symptoms were tingling under the electrodes, headache, scalp dryness, difficulties concentrating, sleepiness, and transient redness at the stimulation site. Only 5 events in total were rated as severe, and none required medical treatment beyond brief telephone triage or led to permanent discontinuation of stimulation (See Table 2).

No stimulation-related serious adverse events (such as seizures, syncope, or clinically significant skin lesions) were observed. Systematic monitoring also included the emergence of manic/hypomanic episodes; none were detected during the trial. One participant triggered an acute suicide-risk alert and was urgently referred to their treating psychiatrist; the episode was managed with brief hospitalization and subsequent stabilization, without the need to terminate the HB-tDCS program.

Overall, the semi-supervised home-based protocol showed high feasibility, with good session completion, manageable technical demands, and a safety profile consistent with previous tDCS literature in depression.

### Clinical outcomes

At baseline, participants showed moderate-to-severe depressive symptoms, with mean scores of 30.24 (SD = 8.20) on the MADRS, 40.70 (SD = 10.12) on the BDI-II, and 25.39 (SD = 6.68) on the QIDS-SR16.

Across the 6-week intervention, we observed significant reductions in depressive symptomatology on all three scales (See Table 3). MADRS scores decreased from 30.24 (SD = 8.20) to 22.91 (SD = 10.19), corresponding to a large within-subject effect (W = 526.50, z = 4.91, p < .001, rank-biserial r = 0.994). BDI-II scores decreased from 40.70 (SD = 10.12) to 35.79 (SD = 14.02), with a moderate effect size (W = 440.50, z = 2.86, p = .002, r = 0.570). QIDS-SR16 scores declined from 25.39 (SD = 6.68) to 21.58 (SD = 7.49; W = 420.00, z = 3.37, p < .001, r = 0.694).

Using a priori thresholds based on percentage change from baseline, 16 of the 33 completers (48.5%) met criteria for clinical response (≥ 25% reduction in MADRS), including 5 patients (15.2%) who achieved a ≥ 50% reduction. The remaining 17 participants (51.5%) were classified as non-responders. Regarding remission, 1 participant (3.0%) reached full remission and 13 (39.4%) achieved partial remission (MADRS in the mild range), yielding 14 patients (42.4%) with at least some degree of symptomatic remission at post-treatment.

Exploratory regression analyses indicated that greater baseline depressive severity (MADRS and QIDS-SR16 scores; MADRS: β = 0.418, p = 0.005; QIDS-SR16: β = 0.415, p = 0.007) and older age (β = 0.393, p = 0.007) were associated with smaller relative improvements in MADRS (i.e., less negative MADRS% change), with the final model explaining roughly 50% of the variance in percentage symptom change (adjusted R^2^ ≈ 0.44).

#### 3.0.1 Cognition and quality of life

Montreal Cognitive Assessment (MoCA) scores increased from 22.76 (SD = 4.66) at baseline to 23.48 (SD = 4.65) post-treatment (t(32) = − 1.81, p = .040, Cohen’s d = 0.32), suggesting an improvement in global cognitive performance over the 6-week HB-tDCS protocol.

Quality of life, assessed with the Q-LES-Q-SF, showed no significant changes from pre- to post-treatment (pre = 36.48 (SD10.11); post = 37.24 (SD = 11.60); t(32) = − 0.53, p = .300, Cohen’s d ≈ 0.09).

### Resting state EEG

**Figure 2:**
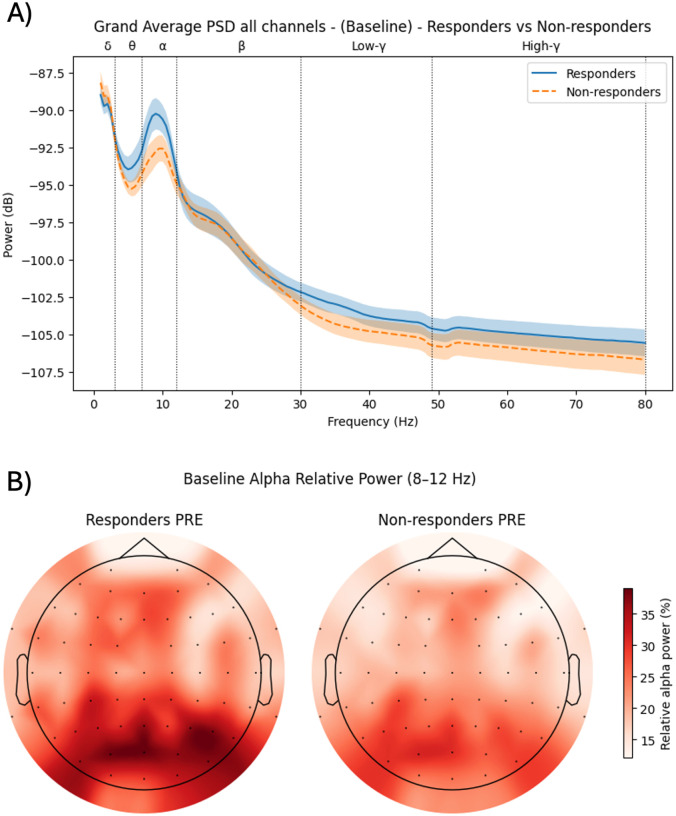
Resting-state EEG results. A) Mean power spectral density (PSD) at baseline during resting state, averaged across all electrodes (responders vs. non-responders). B) Baseline alpha-band relative spectral power (rSP) scalp topographies for responders (left) and non-responders (right). Colors indicate the spatial distribution of relative power (warmer colors = higher rSP). Although the primary region-of-interest analysis focused on F3, whole-scalp maps are shown to contextualize the spatial distribution of alpha-band rSP..

At the whole-head level, responders showed a tendency toward higher global alpha power compared with non-responders at baseline. The peak of the mean alpha power spectrum was − 90.24 dB in responders versus − 92.57 dB in non-responders, consistent with a descriptively stronger alpha rhythm in those who later improved clinically (see Fig. 2a).

However, independent-samples tests on absolute power (aSP) and relative power (rSP) in the alpha band at the global level did not reach conventional significance (all p > .05, with p values in the trend range for some contrasts). Dominant peak frequency (DPF) and dominant peak amplitude (DPA) in the 8–12 Hz range also did not differ significantly between groups, either globally or when restricted to the left frontal ROI.

Focusing on the left dorsolateral prefrontal cortex (electrode F3), the same qualitative pattern was observed: responders tended to show slightly higher absolute and relative alpha power at baseline compared with non-responders (see Fig. 2b). A one-tailed comparison, based on the a priori hypothesis of higher frontal alpha in responders, yielded a significant group difference for relative alpha power at F3 (rSP; p = .038), whereas absolute power and peak-based measures (DPF, DPA) remained non-significant. Thus, baseline left frontal alpha power emerged as a putative, but still modest, candidate marker of subsequent clinical response.

### TMS evoked Potentials (TEP) Components

TMS-EEG analyses focused on TMS-evoked potentials (TEPs) recorded over the left DLPFC at the anxiosomatic (Som) and dysphoric (Dys) targets as defined in Methods. We examined early positive components (P30, P60), a later positive component (P180), negative components (N45, N100), and, in exploratory analyses, global mean field amplitude (GMFA-AUC). Clinical response (responder vs non-responder) was entered as a between-subject factor.

#### Positive TEP components

**Figure 3:**
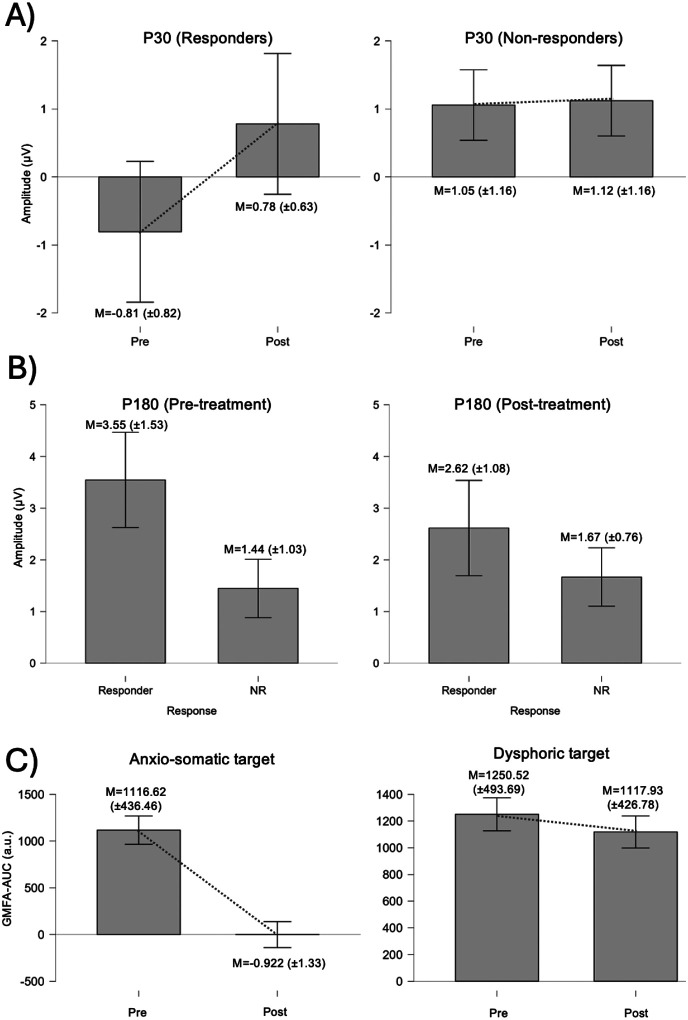
TMS-EEG results. A) P30 amplitude at the anxiosomatic (Som) target. Two panels show pre- vs. post-treatment comparisons within each response group: responders (left) and non-responders (right). B) P180 amplitude at the anxiosomatic (Som) target. Two panels show between-group comparisons (responders vs. non-responders) at each time point: pre-treatment (left) and post-treatment (right). C) GMFA-AUC shown pre- and post-treatment for the whole sample. The left panel corresponds to the anxiosomatic (Som) target and the right panel to the dysphoric (Dys) target.

All predefined TEP components were analysed at both stimulation targets using the same repeated-measures framework; here we highlight the effects that reached statistical significance and summarize null findings for the remaining components. When clinical response was included as a between-subject factor, a repeated-measures ANOVA on P30 amplitude at the anxiosomatic (Som) target revealed a significant main effect of Time, F(1, 13) = 7.87, p = .015, η^2^G = .137, as well as a significant Time × Response interaction, F(1, 13) = 6.68, p = .023, η^2^G = .119. This pattern indicates that P30 amplitude changed from pre- to post-treatment and that the magnitude and/or direction of change differed between responders and non-responders (see Fig. 3a). Post hoc comparisons showed that responders exhibited a negative P30 at baseline that shifted toward positive values post-treatment (M_pre = − 0.81, M_post = 0.78; paired t(4) = 3.01, p = .040), whereas non-responders remained positive and showed no pre–post change (M_pre = 1.06, M_post = 1.12; paired t(9) = 0.20, p = .846); responders and non-responders differed at baseline (Welch’s t(11.11) = 3.60, p = .004) but not post-treatment (p = .475). In addition, there was a significant between-subjects effect of Response, F(1, 13) = 5.00, p = .044, η^2^G = .221, indicating overall amplitude differences between groups, with non-responders showing more positive P30 values than responders across time.

For the P180 component at the Som target, the repeated-measures ANOVA did not show a significant main effect of Time, and the Time × Response interaction remained at trend level (p = .10). However, there was a robust between-subjects effect of Response (p < .001), indicating that responders exhibited consistently larger P180 amplitudes than non-responders across pre- and post-treatment assessments. Thus, while P180 did not show a clear pre–post change, responders exhibited larger P180 amplitudes than non-responders already at baseline (M = 3.55, SD = 1.53 vs. M = 1.45, SD = 1.03; Welch’s t(16.99) = 3.87, p = .002, Holm-adjusted) and this difference remained at post-treatment (M = 2.62, SD = 1.08 vs. M = 1.67, SD = 0.76; Welch’s t(17.56) = 2.44, p = .025; see Fig. 3b).

At the dysphoric (Dys) target, no significant pre–post effects or Time × Response interactions were observed for the positive TEP components (P30, P60, P180), and no reliable amplitude differences emerged between responders and non-responders.

#### Negative TEP components

For the negative TEP components, analyses focused on the N45 and N100 components at both the anxiosomatic (Som) and dysphoric (Dys) targets.

For N45, repeated-measures ANOVAs did not reveal significant main effects of Time or Time × Response interactions at either target (all p > .10), indicating no robust pre–post modulation associated with the intervention. However, at the anxiosomatic (Som) target, a significant between-subjects effect of Response was observed, F(1, 20) = 6.54, p = .019, indicating overall differences in N45 amplitude between responders and non-responders that were stable across pre- and post-treatment assessments with responders showing more negative N45 amplitudes than non-responders (Pre: −2.81 vs − 0.85; Post: −4.22 vs − 0.30). No corresponding group differences were detected at the dysphoric (Dys) target.

For N100, repeated-measures ANOVAs similarly showed no significant main effects of Time and no Time × Response interactions at either target (all p ≥ .16). In addition, no significant between-subjects effects of Response were observed for N100 at either the Som or Dys target (all p ≥ .19), indicating comparable N100 amplitudes between responders and non-responders across time.

Taken together, these results suggest that, within the present sample, negative TEP components did not exhibit clear treatment-related modulation over time. Aside from a stable group difference in N45 amplitude at the anxiosomatic target, inhibitory-related TEP components did not reliably differentiate responders from non-responders.

#### Global measures

Exploratory analyses of global mean field amplitude area under the curve (GMFA-AUC) in the whole sample showed target-specific effects. At the dysphoric target, GMFA-AUC did not change significantly from pre- to post-treatment (Wilcoxon signed-rank test: W = 350, z = 1.61, p = .055; effect size r = 0.33), suggesting that global cortical excitability remained stable at this stimulation site.

In contrast, stimulation of the somatic target was associated with a significant pre–post increase in GMFA-AUC (Wilcoxon signed-rank test: W = 378, z = 4.54, p < .001; effect size r = 1.00), indicating a marked enhancement of global cortical reactivity following treatment, but totally unrelated to treatment response (see Fig. 3c).

## 4 Discussion

In a naturalistic cohort of patients with treatment-resistant depression (TRD), a semi-supervised home-based tDCS (HB-tDCS) program proved feasible, well tolerated, and associated with significant reductions in depressive symptoms after 6 weeks.

Daily dosing over six weeks was implementable at home with training, impedance control, and tele-follow-up. Although restarts due to high impedance and temporary interruptions were relatively frequent, they primarily reflect device safeguards and quality control rather than clinically limiting events; most sessions ultimately completed. From a service perspective, the model leverages asynchronous monitoring plus brief weekly contacts, keeping clinician time modest while maintaining oversight. These data converge with the growing HB-tDCS literature showing high acceptability and operational feasibility under remote supervision.

Notably, we delivered stimulation 7 days per week with no weekend breaks, a more intensive cadence than most tDCS depression protocols, which may have increased cumulative dose over a shorter calendar period and is therefore important to consider when comparing feasibility, tolerability, and outcomes across studies.

Impedance-related interruptions are a known practical issue in at-home tDCS, where impedance variability tends to be higher than in-clinic administration and has been linked to tolerability/safety signals in some datasets (Vogelmann et al., 2025). Our findings are consistent with the view that most “technical” events in home settings arise from the electrode–skin interface (contact quality, drying, movement) and can be mitigated through structured training and brief remote support.

This aligns with other home-delivered neuromodulation programs that emphasize usability and monitoring/support to maintain adherence and safety, including fully remote home tDCS protocols with real-time supervision, as in the Flow trial (Woodham et al., 2024), and physician-directed at-home neuromodulation with high compliance in a pivotal sham-controlled study (eCOT-AS / ProlivRx) (Carpenter et al., 2025).

The adverse-event profile mirrors prior tDCS experience: headache, tingling under electrodes, sleepiness, concentration difficulties, skin dryness, and redness were the most common and mild; importantly, no stimulation-related SAEs occurred. Two AE-triggered calls (0.1%) and rare escalation (psychological support in 0.3%; psychiatrist referral in 0.07%) suggest that structured triage pathways are adequate for remote programs. While our data support a favorable safety profile, continued surveillance remains essential when scaling beyond research settings.

The magnitude of symptom changes over 6 weeks—a 24% reduction on the MADRS, with 48.5% meeting response criteria, aligns well with the existing HB-tDCS literature despite the absence of a control group. Baseline severity in our cohort (MADRS ~ 30) was comparable to that reported in the Neurolief pivotal sham-controlled study (MADRS ~ 30) and higher than in the fully remote Flow trial (MADRS ~ 24), which should be considered when comparing response rates across studies (Carpenter et al., 2025; Woodham et al., 2024). Recent randomized controlled trials in MDD have reported higher response rates with active tDCS than sham (approximately 40–60% vs. 20–30%, respectively) (Brunoni Andre R. et al., 2017; Razza et al., 2020; Woodham et al., 2024) Notably, differences in baseline severity, degree of treatment resistance, and trial design (fully remote vs. in-clinic visits; intensity and supervision) likely contribute to between-study variability in outcomes. Meta-analytic estimates of antidepressant efficacy for tDCS typically fall in the small-to-moderate range (standardized mean differences ~ 0.4–0.7), depending on sample characteristics and stimulation dose (Moffa et al., 2020; Nikolin et al., 2023). In this context, our observed effect sizes fall within or exceed these benchmarks, suggesting that intensive daily dosing over 6 weeks, delivered alongside ongoing pharmacotherapy in a naturalistic TRD sample, can yield clinically meaningful benefit comparable in magnitude to that reported in controlled efficacy trials. The convergence between our real-world outcomes and published RCT data further supports HB-tDCS as a viable treatment option for TRD when delivered with appropriate training, quality control, and remote supervision. Our response thresholds (≥ 25% for partial response; ≥50% for response) offer a clinically interpretable frame for future controlled work. The discrepancy between clinician-rated and self-report outcomes warrants attention in trial design (e.g., prespecifying a single primary endpoint and using the others as secondary/triangulation measures).

HB-tDCS reduces travel and chair time, potentially improving engagement in TRD populations with functional impairment. The operational protocol used here, appears sufficient to achieve high session completion while keeping clinician time modest. Pragmatically, future programs should prespecify adherence thresholds (e.g., ≥ 36/42 sessions), establish make-up rules for missed days, and standardize skin preparation to reduce impedance-related restarts. Embedding brief digital checklists after each session provided actionable signals for follow-up without saturating clinician workload, supporting the feasibility of scalable, semi-supervised delivery in routine-care pathways.

Crucially, this pragmatic implementation was complemented by mechanistic assessments that begin to clarify who is most likely to benefit and why. Baseline TMS-evoked potentials (TEPs) over the anxiosomatic DLPFC differentiated clinical responders from non-responders: the P30 component showed a significant Time × Response interaction, and P180 exhibited robust baseline between-group differences, with responders displaying consistently higher amplitudes. Interpreted mechanistically, P30 is commonly linked to early cortical excitability and glutamatergic neurotransmission in prefrontal circuits (Premoli et al., 2014). The differential modulation of P30 across treatment suggests that clinical benefit may depend on the capacity for neuroplastic change during repeated daily stimulation, consistent with models implicating altered prefrontal excitability and excitatory–inhibitory balance in depression and its cognitive-control deficits (Hadas et al., 2019; Voineskos et al., 2021). This aligns with emerging evidence from TMS-EEG in rTMS/theta-burst paradigms indicating that baseline TEP profiles, and their modulation over treatment, can predict antidepressant outcomes (Eshel et al., 2020; Strafella et al., 2023).

In contrast, P180, often interpreted as reflecting later recurrent activity and longer-range network integration, did not show a clear treatment-related shift, suggesting a more stable trait-like marker of network integrity (Premoli et al., 2014). Higher baseline P180 in responders may reflect relatively preserved prefrontal network communication, positioning patients to benefit from increased DLPFC excitability and downstream top-down regulation of mood-related circuits. This interpretation converges with network-based targeting findings in depression showing that stimulation sites and baseline connectivity profiles, particularly with the subgenual cingulate, relate to antidepressant outcomes (Fox et al., 2012; Siddiqi et al., 2020; Siddiqi and Fox, 2024; Williams, 2017, 2016).

Notably, the predictive value of TEPs was target-specific: effects were observed at the anxiosomatic DLPFC target but not at the dysphoric target, consistent with evidence that different DLPFC subregions map onto partially distinct symptom dimensions and circuits (Siddiqi et al., 2020; Williams, 2017, 2016). This dissociation suggests that modulation of anxiosomatic circuitry may be a key pathway through which HB-tDCS produces broader antidepressant effects in TRD, and motivates future studies testing whether baseline TEPs predict dimension-specific symptom change and whether neurophysiologyinformed targeting improves response rates.

Resting-state EEG provided convergent evidence: responders showed higher baseline left-frontal alpha (F3) relative power, consistent with literature suggesting that frontal EEG features can function as predictors of neuromodulation outcomes (Roelofs et al., 2021; Romero-Marín et al., 2026b; Widge et al., 2019). The convergence between resting EEG (alpha) and TMS-EEG (P30/P180) supports multimodal neurophysiological profiling as a feasible “precision layer” that can be embedded within pragmatic HB-tDCS programs. If replicated in independent samples and RCTs, these markers could inform patient selection, guide personalized dosing or augmentation strategies (e.g., pairing stimulation with cognitive training or plasticity-enhancing pharmacological approaches), and accelerate the transition from one-size-fits-all protocols to precision neuromodulation tailored to individual circuit-level profiles.

This study present strength and limitations. Strengths include (i) a consecutive TRD cohort implementing a daily at-home regimen, (ii) comprehensive feasibility logging (completed/omitted sessions, impedance restarts, interruptions, support contacts), and (iii) convergent outcomes across clinician-rated and self-reported measures. The home-based setting enhances ecological validity and informs scale-up logistics.

Limitations are represented first by the pre–post design without a control arm, that precludes to make strong claims about causal inference on the efficacy of the stimulation. Second, the sample size, though larger than preliminary pilots, remains modest. Third, neurophysiology and neuroimaging were preliminary/not analyzed here, limiting mechanistic interpretation. Fourth, assessment burden likely contributed to attrition and missingness at post-treatment in a minority of cases. Finally, the feasibility metrics (e.g., impedance restarts) partly reflect conservative device safety logic and may not generalize to platforms with different thresholds. Last, naturalistic conditions introduce time-varying influences (e.g., major life events and medication changes) that can modulate mood and cognition, inflate variance, and attenuate observed effect sizes. In particular, most patients were receiving polypharmacy, and converging evidence indicates that concomitant psychotropic medication can modulate tDCS-induced plasticity, sometimes enhancing and sometimes diminishing neuromodulatory effectsdepending on drug class, dose, receptor profile, and baseline neurophysiology (Kuo and Nitsche, 2021; Nitsche et al., 2006). Because much of the drug–tDCS interaction literature comes from motor-cortex studies in healthy participants, extrapolation to prefrontal stimulation in TRD should be made cautiously (Kuo and Nitsche, 2021). Prospective designs should (a) capture time-varying covariates (life-events diary; medication changes), (b) prespecify sensitivity analyses controlling for such covariates, and (c) consider EMA (ecological momentary assessment) to improve signal-to-noise between visits.

To overcome this limitation future studies must employ a randomized, sham-controlled trial design, with prespecified primary endpoint, blinding integrity checks, and covariate capture for life events and medication would strengthen inference. Cost-effectiveness and implementation studies should evaluate training time, staff oversight load, and uptake in routine services. Hybrid effectiveness-implementation designs could test refined assessment batteries (short forms/CAT), automated impedance coaching, and adaptive dosing (e.g., session number or spacing tailored to early trajectories). If neurophysiology remains in scope, focusing on parsimonious markers (e.g., pre-treatment P30/N45) and clinically actionable cut-offs may improve translational value.

To conclude, a semi-supervised home-based tDCS program for TRD is feasible, well tolerated, and associated with meaningful symptom reductions over six weeks in a naturalistic cohort. The operational recipe, patient/companion training, impedance-gated delivery, and light-touch tele-supervision, supports high session completion and minimal escalation. While these findings align with the emerging HB-tDCS literature, controlled trials are needed to confirm efficacy, clarify the role of concomitant treatments, and optimize assessment strategies for real-world deployment.

These findings provide strong clinical support for HB-tDCS as a feasible, well-tolerated, and practically deployable adjunct treatment for TRD, and they highlight the added value of biomarkers to identify patients most likely to respond and to guide patient selection in real-world services.

## Figures and Tables

**Table 1:** Participant’s sociodemographic characteristics. Age, years since MDD diagnosis and baseline MDD severity scores measured with MADRS are presented as mean (SD); all other variables are presented as percentages.

Variable	n = 40
Age (years)	54.20 (10.07)
Sex	
Male	25.00%
Female	75.00%
Educational level	
Primary (≤ 6 years)	27.50%
Secondary (7–9 years)	12.50%
Medium grade (10–12 years)	22.50%
Top Grade (13–14 years)	12.50%
University studies (≥ 15 years)	25.00%
Depression characteristics	
Years that have been diagnosed	15.45 (9.46)
Baseline MDD severity scores	30.24 (8.20)

**Table 2:** Adverse effects recorded during treatment. The total number of sessions in which each adverse effect was reported is shown according to severity. Percentages are calculated based on the total number of study sessions (n = 1.219).

Adverse effects	Mild	Moderate	Severe	Total	%
Headache	63	41	0	104	*8.5*
Tingling	67	49	2	118	*9.7*
Sleepiness	2	17	0	19	*1.6*
Concentration problems	2	41	3	46	*3.8*
Scalp redness/burns	10	10	0	20	*1.6*
Dryness	12	31	0	43	*3.5*
*Total*	*156*	*189*	*5*	*350*	*28.7*

**Table 3:** Wilcoxon signed-rank tests for pre–post comparisons. For all analyses, the alternative hypothesis specified that pre-treatment scores (Measure Pre) were greater than post-treatment scores (Measure Post) (one-tailed tests). Effect sizes are reported as rank-biserial correlations.

Measure Pre		Measure Post	Statistic	z	p	Effect Size	SE Effect Size
MADRS pre	-	MADRS post	526.500	4.908	< .001	0.994	0.200
BDI pre	-	BDI post	440.500	2.859	.002	0.570	0.197
QIDS pre	-	QIDS post	420.000	3.371	< .001	0.694	0.203
